# Myocardial fibrosis is not associated with reduced quality of life in patients with dilated or hypertrophic cardiomyopathy

**DOI:** 10.1186/1532-429X-13-S1-P292

**Published:** 2011-02-02

**Authors:** Razi Khan, David Massel, David Scholl, John Stiratt, Gerald Wisenberg, R Terry Thompson, Frank Prato, Derek Boughner, Maria Drangova, James A White

**Affiliations:** 1 Division of Cardiology, Department of Medicine, London Health Sciences Centre, London, ON, Canada; 2 Imaging Research Laboratory - Robarts Research Institute, University of Western Ontario, London, ON, Canada; 3 Lawson Health Research Institute, University of Western Ontario, London, ON, Canada

## Background and objectives

Patients with Dilated Cardiomyopathy (DCM) and Hypertrophic Cardiomyopathy (HCM) frequently demonstrate non-ischemic pattern myocardial fibrosis (MF) on delayed enhancement magnetic resonance imaging (DE-MRI). The clinical significance of this finding with respect to impact on Quality of Life (QOL) is poorly understood. In this study we identify the prevalence of MF in patients with HCM and DCM and assess its relationship with standardized measures of QOL.

## Methods

One-hundred and seven consecutive patients with either DCM (n=50) or HCM (n=57) referred for MRI evaluation were identified. DCM was defined as a LVEF <40% and no significant coronary disease by angiography. HCM was defined by standard echocardiographic criteria. All patients completed both the Minnesota Living With Heart Failure (MLWHF) and SF-12 QOL questionnaires at time of MRI. A standard DE-MRI protocol was performed 10 minutes following the administration of intravenous gadolinium (Gadovist®, Bayer Inc). DE images were both visually scored using a validated 68 sub-segment segment model and quantitatively analyzed for signal enhancement (threshold >3SD above reference myocardium) using validated commercial software.

## Results

Mean age for DCM and HCM groups was 59 (±13.4) and 53 (±12.1) years respectively with a mean ejection fraction of 34.7 ±16.9% and 74.2 ±10.8 % respectively. The respective mean NYHA class of the 2 groups was 2.0 ±0.9 and 1.4 ±1.1. The prevalence of any MF on visual scoring was 64% in the DCM group and 82% in those with HCM. By quantitative evaluation there was significantly more MF detected in patients with HCM than in DCM (19.2% vs. 13.1% of LV mass (p=0.008)). QOL, as assessed by MHLWF, was not significantly different in patients with any MF versus those without for either the DCM group (39.9 vs. 26.1, p=0.067) or HCM group (28.7 vs. 26.1, p=0.45). The SF12 physical and mental scores were also similar for both the DCM group (46.4 vs. 50.2 (p=0.25), and 46.2 vs. 44.4 (p=0.83), respectively) and HCM group (40.4 vs. 45.5 (p=0.36), and 46.2 vs. 44.4 (p=0.83), respectively). There was no correlation found between the quantitative volume of MF and any QOL score in either patient group.

## Conclusion

MF is common in patients with DCM and HCM. However, its presence and severity is not correlated with reductions in QOL relative to patients without MF. Prospective studies to evaluate the impact of MF on the natural history of disease progression and interim change in QOL are underway.

